# The Properties of Adaptive Walks in Evolving Populations of Fungus

**DOI:** 10.1371/journal.pbio.1000250

**Published:** 2009-11-24

**Authors:** Sijmen E. Schoustra, Thomas Bataillon, Danna R. Gifford, Rees Kassen

**Affiliations:** 1 Department of Biology, University of Ottawa, Ottawa, Ontario, Canada; 2 Center for Advanced Research in Environmental Genomics, University of Ottawa, Ottawa, Ontario, Canada; 3 Institute of Biology, Bioinformatics Research Centre, Aarhus University, Aarhus, Denmark; 4 INRA, UMR DIAPC, Montpellier, France; University of Edinburgh, United Kingdom

## Abstract

The rarity of beneficial mutations has frustrated efforts to develop a quantitative theory of adaptation. Recent models of adaptive walks, the sequential substitution of beneficial mutations by selection, make two compelling predictions: adaptive walks should be short, and fitness increases should become exponentially smaller as successive mutations fix. We estimated the number and fitness effects of beneficial mutations in each of 118 replicate lineages of *Aspergillus nidulans* evolving for approximately 800 generations at two population sizes using a novel maximum likelihood framework, the results of which were confirmed experimentally using sexual crosses. We find that adaptive walks do indeed tend to be short, and fitness increases become smaller as successive mutations fix. Moreover, we show that these patterns are associated with a decreasing supply of beneficial mutations as the population adapts. We also provide empirical distributions of fitness effects among mutations fixed at each step. Our results provide a first glimpse into the properties of multiple steps in an adaptive walk in asexual populations and lend empirical support to models of adaptation involving selection towards a single optimum phenotype. In practical terms, our results suggest that the bulk of adaptation is likely to be accomplished within the first few steps.

## Abstract 2

A novel method to infer the number and fitness effect of beneficial mutations reveals that the bulk of adaptive evolution is attributable to a few mutations with variable effects on fitness.

## Author Summary

Adaptation is one of the least understood processes in biology because it relies on beneficial mutations, which are often too rare to study. We developed a method to infer the number and size of beneficial mutations substituted during adaptation, a process called an *adaptive walk*, and used this to test predictions about the properties of adaptive walks in experimental populations of fungus. Our work shows that, in contrast to the gradualist view of adaptation dominant since the 1930s, adaptive walks tend to be fast and short, with beneficial mutations of large effect substituted first, followed by those of smaller effect.

## Introduction

The rate and extent of adaptive evolution over long time periods depends ultimately on the sequential substitution of beneficial mutations by natural selection, a process termed an *adaptive walk*. Although recent work with microbial populations has shed light on the properties of a single bout of adaptation [1]–[7], no empirical data exist on the properties of multiple steps in an adaptive walk. Here, we present what is, to the best of our knowledge, the first comprehensive empirical results on the distribution of fitness effects among beneficial mutations for multiple steps of an adaptive walk and confront these with theoretical predictions concerning the number and fitness effect of mutations involved in adaptation.

The dominant view championed since the 1930s by Fisher through his geometric model of adaptation has been that adaptive walks are driven by the substitution of many mutations with exceedingly small fitness effects [8]. More recent theoretical work gives a substantially different picture. Adaptation can be modeled as a sequence of moves or steps in either phenotype [9]–[12] or DNA sequence [8],[13]–[17] space, via mutation, that increase fitness relative to the current wild type. Adaptation stops once a population becomes fixed for a genotype where all neighboring genotypes accessible by mutation have lower fitness. Provided the wild type is fairly well adapted to current conditions, beneficial mutations in both models can be viewed as draws from the right tail of a distribution of fitness effects [12], and extreme value theory (EVT) can be used to make predictions about the distribution of fitness effects among beneficial mutations available to and fixed by selection [18]–[21]. Note that although these models allow for genetic drift through the stochastic loss of mutations when rare, they assume sufficiently strong selection that deleterious mutations are always effectively removed and “weak” mutation, meaning that beneficial mutations occur sufficiently rarely that clonal interference, the competition that results from beneficial mutants arising in different lineages that have escaped drift but have yet to fix, can be neglected. These so-called “strong selection–weak mutation” assumptions are no longer strictly appropriate when the mutation supply rate, which is the product of population size (*N*) and mutation rate (*μ*), is high, because clonal interference becomes increasingly important [4],[22]–[24]. Nevertheless, these models provide a useful starting point for thinking about the properties of adaptive walks.

With these caveats in mind, models of adaptation make three compelling predictions about the nature of the distributions of mutational effects over the course of an adaptive walk. First, the combined effects of genetic drift, which tends to eliminate small-effect mutations, and clonal interference transforms the typically L-shaped distribution of fitness effects available to selection into a distribution of fitness effects among fixed mutations that is unimodal with a substantially higher mean [9]. Second, the pattern of fitness gains over the course of an adaptive walk depends on the distribution of fitness effects among available mutations. If this distribution is exponential (Gumbel domain of attraction in EVT), or nearly so, the mean fitness effect of each successive mutation fixed will be identical throughout an adaptive walk. Alternatively, if the distribution is truncated on the right (the Weibull domain in EVT), for example as predicted in an adaptive landscape—which describes how fitness changes as a function of either genotype or phenotype—involving selection towards a single phenotypic optimum [21], adaptation is characterized by a pattern of decreasing fitness gains at each step [17]. Third, adaptive walks tend to be short, relative to the total number of beneficial mutations available. Precise predictions of the number of steps taken are difficult to make because they depend on the ruggedness of the adaptive landscape and the stringency of selection [15]. A lower bound on the expected number of mutations substituted on rugged landscapes (technically, a random landscape with no autocorrelation) when the fittest beneficial mutation available always fixes has been suggested to be 1.7 mutations on average [14],[25]. This number is expected to be only modestly higher when landscapes are smoother and selection is less stringent, such as when drift can lead to the loss of even large-effect mutations on occasion; simulations suggest the number of substitutions under these conditions is on the order of three to five [26].

**Figure 1:**
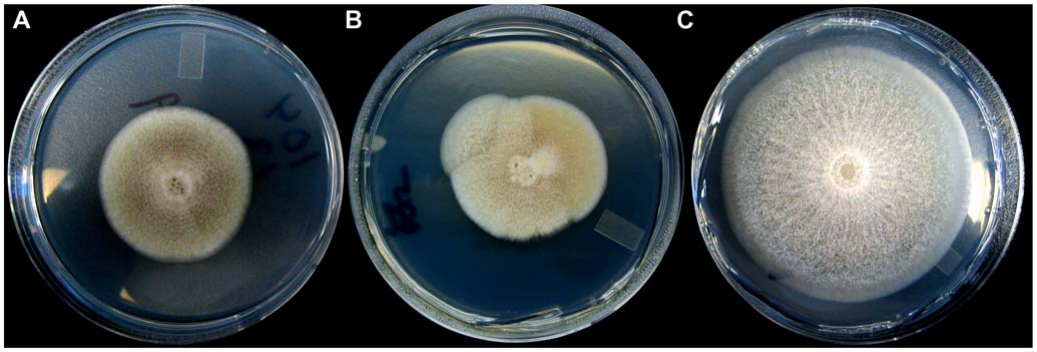
Five-day-old *A. nidulans* growing on solid medium. (A) Ancestral genotype used to initiate the experiment; (B) as in (A), but with at least two independent beneficial mutations that have arisen during a single cycle of growth; (C) an evolved lineage from the end of the experiment. Note the difference in colony diameter between the ancestral and evolved lineages.

A comprehensive empirical test of these predictions requires following the fitness trajectories of a large number of replicate evolving lineages over many generations and estimating the number and effect size of the mutations fixed along the way. What experimental data exist focus exclusively on the number of mutations fixed, but not their effect size during adaptive walks in a limited number (usually one to three) of replicate lineages [27]–[32]. We developed a novel maximum likelihood (ML) framework (see Box 1 and Text S1) to infer the number and size of adaptive mutations that occurred during adaptation over 800 generations in 118 replicate lineages founded by a single genotype of the filamentous fungus *Aspergillus nidulans*. This species forms spatially structured colonies on solid surfaces, making it relatively easy to detect beneficial mutations (Figure 1). We founded all lineages from a single ancestral genotype and propagated replicate lineages by serial transfer every 5 d (which corresponds to ∼80 generations) to fresh medium, bottlenecking half the lineages to approximately 50,000 nuclei and the other half to approximately 500 nuclei at each transfer. The ancestral genotype contains a mutation conferring resistance to the fungicide fludioxonil that is known to be costly under the growth conditions used here, and previous work has shown that a variety of genetic routes to adaptation are available to this genotype [33],[34]. The results of the ML procedure were validated experimentally by estimating the number of segregating loci in the F1 progeny of sexual crosses between selected evolved lineages and the ancestor using the Castle-Wright estimator [30],[35].

**Box 1. Maximum likelihood framework for estimating the number and effect size of beneficial mutations fixed**

Assumptions of the model

We assume a strictly clonal population growing exponentially at rates that do not vary in time or depend on the frequencies of other clones. We assume that during propagation, clones are serially transferred to fresh medium so there is no density dependence. We assume that the minimum population size during each bottleneck is large enough to not affect the trajectories of allele frequencies between bottlenecks (see Text S1).

Derivation of the likelihood function

In general, we wish to estimate a vector of Malthusian parameters *r* = (*r*_1_, *r*_2_,…, *r_n_*) and times of origin *t* = (*t*_1_, *t*_2_,…, *t_n_*) of up to *n* clones (*n*−1 beneficial mutants plus the ancestral clone) from data consisting of fitness estimates (observations) collected at *k* spaced time points during the adaptation of a single population. The likelihood of the data *D* = (*w*_1_, *w*_2_,…, *w_k_*) is then
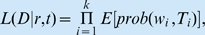
(1)where *E*[ . ] is the expectation over all possible clones present at each time point in the population assayed and *prob*(*w_i_*,*T_i_*) denotes the probability of observing a fitness *w_i_* at time point *T_i_* given a collection of *n* clones with associated fitness *r*_1_, *r*_2_,…, *r_n_*. The model is very general and allows for clonal competition and could readily be extended to include a stochastic component for extreme bottlenecking or various forms of density dependence.

Parameter estimation and hypothesis testing

We maximize numerically this likelihood with respect to *r* and *t* (see Text S1) and thereby obtain ML estimates of the fitness effects of new mutations spreading in each population. The fitness trajectory of each lineage (taken as data) is fitted using sequential models *M*_1_, *M*_2_,…, *M_n_* assuming that 1, 2,…, *n* clones with different fitness *r*_1_, *r*_2_,…, *r_n_* are spreading in the population. Akaike's information criterion (AIC) is used to decide which model (*M_n_*), and thus how many beneficial mutations, best fit the data in each population. Note that although the AIC criterion does not ensure that beneficial mutations in the final model have reached strict fixation (frequency = 1), simulations evaluating the time to reach quasifixation (defined as 1−1/(*N_e_ s*); see [58]) and experimental crosses estimating the numbers of mutations fixed in evolved populations (see Table S1 and Text S3) suggest that the ML procedure gives reasonably accurate estimates of both the fitness effect and number of beneficial mutations fixed.

## Results

### Fitness Trajectories

**Figure 2:**
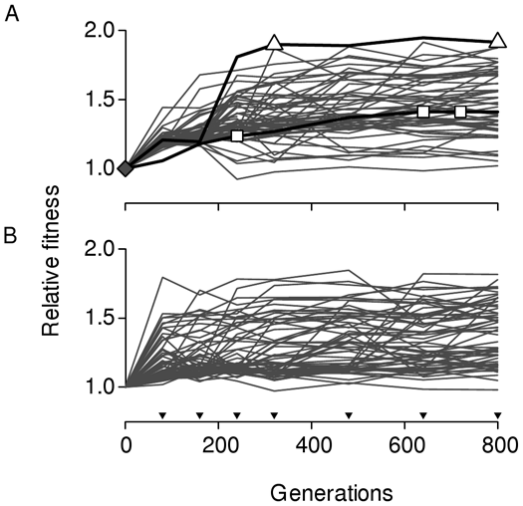
Fitness trajectories. Lines represent the mean of triplicate fitness measures, relative to the ancestor (*w*_ancestor_ = 1.0), of a single lineage propagated at (A) large or (B) small bottleneck size. Standard error bars for each lineage are too small to show. Bold lines in (A) represent lineages chosen at marked time points (open triangles and squares represent the time points sampled; the solid diamond represents the ancestral genotype) to estimate the fraction of beneficial mutations available to selection, shown in Figure 5B. Inverted filled triangles along the *x*-axis in (B) represent times at which fitness was assayed.

The fitness trajectories of all 118 lineages are shown in Figure 2. Mean fitness through time takes the form of a diminishing returns curve in both treatments, with the large bottleneck lineages reaching a higher mean fitness (±standard deviation [s.d.]; 1.48±0.23) on average than the small bottleneck lineages (1.38±0.22) by the end of the experiment (*t*_110_ = 2.10, *p* = 0.038). The variance in fitness among lineages follows a similar diminishing returns pattern but accumulates faster in the small bottleneck treatment (interaction term between time, *t*, and bottleneck size from an analysis of covariance for *t*>0: *F*_1,10_ = 4.97, *p* = 0.05), reaching comparable levels in both treatments by the end of the experiment (*F*_54,60_ = 1.09, *p* = 0.73). These results lend support to the idea that natural selection in populations of different size tends to lead to different effective fitness plateaus [36] and that drift is exaggerated in small populations, the effect of which is to cause more rapid fitness divergence among lineages in the early stages of adaptive evolution [37].

Occasional fitness decreases occurred in both treatments although these were modest in size relative to the initial fitness increase at the first transfer and not sustained over multiple transfers. This variation is likely due to uncontrollable microenvironmental variation in environmental conditions arising during the selection experiment. Alternative explanations include frequency-dependent selection or drift causing a transient increase in the frequency of mildly deleterious mutations. Frequency-dependent selection is a priori unlikely because spatial structure, which facilitates frequency dependent selection by allowing persistent interactions among genotypes [38],[39], was destroyed at each transfer by washing an entire colony off the plate and reinoculating from a well-mixed culture. Furthermore, we did not observe sustained coexistence of distinct colony morphotypes within a population, as would be expected if frequency-dependent selection acted to maintain diversity through mechanisms such as cross-feeding or allelopathy. We can also exclude drift as an explanation, as decreases in fitness between consecutive time points were no more common in the small bottleneck treatment than in the large (small = 13, large = 15; based on pairwise *t*-tests for all lineages between times *t* and *t*+1 using a false discovery rate criterion of α = 0.3 to determine significance), as would be expected if mildly deleterious mutations had a higher probability of increasing in frequency following each transfer.

In previous work, the fungicide-sensitive strain from which the founder of our experiments was derived showed little evidence of adaptation to the same growth conditions used here, suggesting that the sensitive strain resides close to a fitness optimum [40]. Notably, a substantial number of lineages in our experiment have evolved fitness values that remain stable at levels either above or below that of the sensitive ancestor for much of the experiment (Figure S1), implying that these lineages have reached distinct fitness optima.

### A Maximum Likelihood Framework for Estimating the Number and Effect Size of Beneficial Mutations Fixed

Instead of relying on the fitting of fitness trajectories by step functions [28],[31],[33],[41], we developed a rigorous ML method to infer both the number and fitness effect of beneficial mutations segregating at high frequencies in all 118 evolving lineages (Box 1; Text S1). The fitness trajectory of each lineage was used to fit models sequentially assuming 1, 2,…, *n* clones with fitness values *r*1, *r*2,…, *rn* substituting in the population. Comparing the fit of the models to the data allowed us to estimate how many beneficial mutations arose in each lineage and the fitness effects associated with each new mutation. This procedure assumes an exponential model of population growth, and we provide experimental evidence to support this assumption in Figure S2 and Text S2. Independent measures of the number of segregating loci in several evolved lineages derived from experimental crosses [42] (Materials and Methods) with the ancestor independently confirmed the results of the ML analysis (see Table S1 and Text S3). Note that the model does not assume any nesting relationship between clones, meaning that clone *i*+1 does not necessarily arise in the genetic background of clone *i*.

### Distributions of Fitness Effects among Fixed Mutations

**Figure 3:**
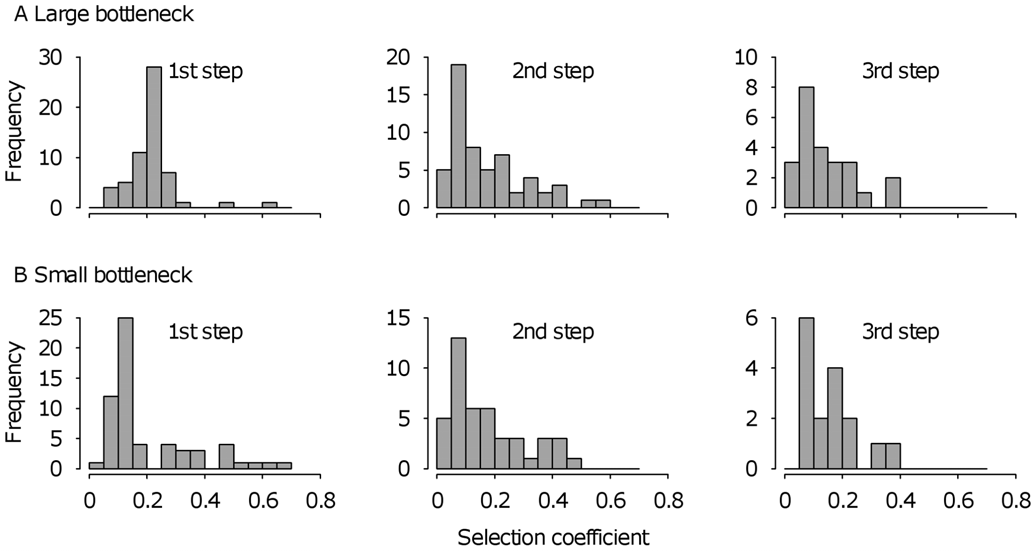
Distributions of fitness effects of mutations fixed in the first, second and third step for (A) the large bottleneck lineages and (B) the small bottleneck lineages.

Figure 3 depicts the distribution of fitness effects among mutations fixed at each step in our experiment. Theory has not been explicit about how the shape of the distribution among fixed mutations is expected to change over the course of an adaptive walk (but see [11]), except to say that the combined effects of drift and clonal competition transform the typically L-shaped distribution of fitness effects among newly arisen mutations into a bell-shaped distribution of fixed fitness effects [4],[9],[19],[43]. Our results shed some empirical light on this issue. All distributions from the large bottleneck treatment are unimodal and positively skewed, as observed previously for a single step [4],[6],[44], however, the shape of the distributions from the small bottleneck treatment are more variable. In particular, the first step differs markedly between treatments (compare the left-most panels of Figure 3A and 3B; permutation test following reference [45]; *n* = 100,000 permutations comparing the absolute value of the difference in coefficients of variation, *d*, between two distributions: *d* = 0.387, *p*<0.0001), the small bottleneck treatment appearing more L-shaped than in the large bottleneck treatment. This result suggests that weaker clonal competition arising from smaller population sizes leads to the fixation of more small-effect mutations and the fixation of mutations with a wider range of effects. Interestingly, the differences between treatments at steps 2 and 3 are not significant (step 2: *d* = 0.059, *p* = 0. 250; step 3: *d* = 0.008, *p* = 0.460), implying that clonal competition becomes less important in shaping the distributions of fixed effects as the population adapts. Further support for this interpretation comes from the fact that significant differences were observed between the first and second steps in the large bottleneck treatment (*d* = 0.353, *p*<0.001), but not between the second and third steps (*d* = 0.149, *p* = 0.098), nor between any of the steps in the small bottleneck treatment (step 1 vs. 2: *d* = 0.026, *p* = 0.380; step 2 vs. 3: *d* = 0.200, *p* = 0.058). Inspection of Figure 3A suggests that this is due to the fixation of smaller-effect mutations in the second step compared to the first step of the large bottleneck treatment, as would be expected if clonal competition becomes weaker as fitness increases.

### The Number of Steps Taken

**Figure 4:**
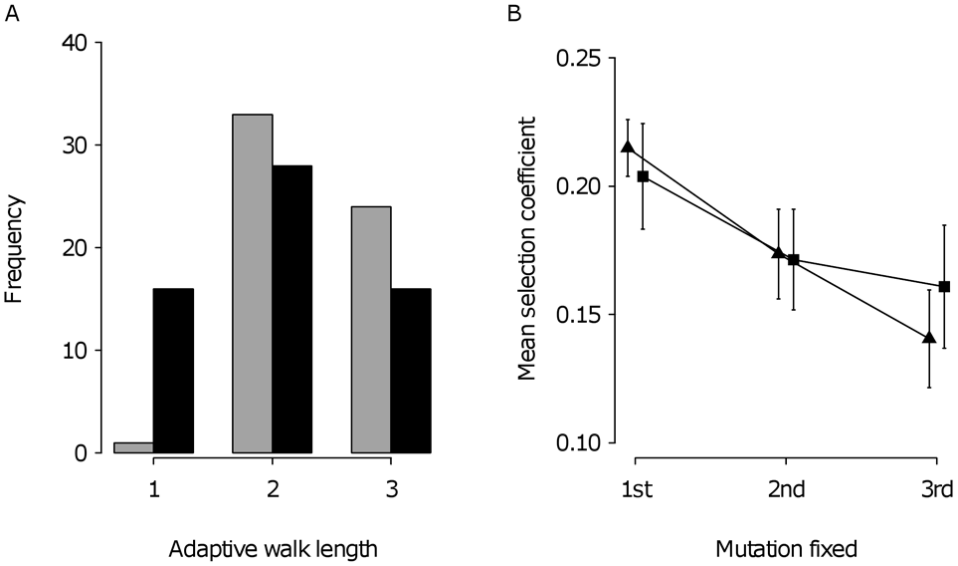
Properties of adaptive walks. (A) Distribution of walk lengths for the experiment. Bars represent observed walk lengths for large (grey) and small (black) bottleneck treatments. (B) Relationship between mean effect size and the number of steps in an adaptive walk for large (diamonds) and small (squares) bottleneck treatments. Error bars indicate ±standard error of the mean. Slopes are significantly negative; see text and Table 1.

Figure 4A shows that adaptive walks tend to be short, with a mean of 2.20 steps for the entire experiment. Large bottleneck lineages fixed more mutations on average (±s.d.; 2.39±0.53) than small bottleneck lineages (2.00±0.74; Wilcoxon rank sum test: *Z* = 3.15, *p* = 0.0016, *n*_large_ = 58, *n*_small_ = 60), consistent with the higher probability of losing beneficial mutations by drift in smaller populations. Mutations restoring sensitivity to fludioxonil were rare in our experiment, being observed in just five of the 118 lineages. In two lineages from the small bottleneck treatment, sensitivity was restored by a single mutation. The remaining three fludioxonil-sensitive lineages (one from the large and two from the small bottleneck treatments) substituted multiple mutations that resulted in restored sensitivity. Removing these lineages from the analysis does not change our results. Thus, the short walks we observed are not merely due to the predominance of back mutations of the resistance mutation (*fld*A1). Moreover, we were able to detect significant genetic (*V_G_*) and genotype-by-environment interaction (*V_GE_*) variation among evolved lineages in both bottleneck treatments (large: *V_G_*: *F*_57,340_ = 5.16, *p*<0.0001, *V_GE_*: *F*_57,340_ = 9.33, *p*<0.0001; small: *V_G_*: *F*_59,342_ = 5.53, *p*<0.0001, *V_GE_*: *F*_59,342_ = 4.80, *p*<0.0001) when grown across a concentration gradient of fludioxonil (Materials and Methods). Thus, although we do not know in detail the identity of the molecular changes responsible for fitness increases, we can be confident that they were achieved through a variety of genetic routes.

### Mean Step Size

The marginal increase in fitness becomes smaller with each successive mutation fixed, the largest steps taken first, followed by consecutively smaller steps (Figure 4B). This relationship is highly significant and independent of bottleneck size (Table 1). Formally, we cannot reject an exponential model as an adequate description of our data (exponent ± 95% confidence interval: −0.309±0. 170), although we cannot reject a linear model either (slope ± 95% confidence interval: −0.038±0.028). Inspection of the variance explained by each model (adjusted *R* ^2^ in Table 1) indicates that the exponential model provides a modestly better fit to the data; however, no stronger inference can be made due to the lack of statistical power associated with observing just three steps. Such a pattern of diminishing fitness effects is consistent with the distribution of fitness effects among beneficial mutations available to selection being right-truncated, that is, it derives from the Weibull domain of attraction of the generalized Pareto distribution [17]. Alternatively, this pattern could arise if large fitness increases are due to the substitution of multiple mutations arising in the same genome and small fitness increases are due to single mutations [46],[47]. Two lines of evidence argue against this hypothesis. First, reducing the supply rate of beneficial mutations should lead to a shallower relationship between fitness increase and number of mutations fixed; however, this was clearly not the case: the relationship between fitness increase and number of mutations fixed in the small bottleneck treatment was negative and indistinguishable from the large bottleneck treatment, despite a mutational supply rate at least two orders of magnitude less. Second, the ML procedure did not consistently underestimate the number of mutations segregating in crosses between evolved lineages and the founder, as would be expected if multiple mutations often hitchhiked together on the same genetic background (see model selection in Text S1and S3, and Table S1).

**Table 1:** Analysis of covariance (Type III) between mean fitness effect at each step and the number of steps taken.

Source	df	MS	*F*	*p*-Value	Adjusted *R* ^2^
**Exponential fit**					0.047
Step	1	6.721	12.223	0.0006	
Bottleneck	1	0.908	1.652	0.1999	
Interaction	1	1.002	1.822	0.1783	
Residuals	255	0.550			
**Linear fit**					0.023
Step	1	0.1350	8.747	0.0034	
Bottleneck	1	0.0005	0.034	0.8544	
Interaction	1	0.0060	0.389	0.5332	
Residuals	255	0.0155			

df, degrees of freedom; MS, mean square.

### Limits on the Number of Steps Taken

The observed short adaptive walks and the negative relationship between the number and size of steps taken are expected if the supply of beneficial mutations declines as a population adapts [10],[13]. We tested this idea directly by characterizing the relationship between the fraction of beneficial mutations available to selection and mean fitness of the genotype from which these mutations were derived. We collected mutants from the original genotype used to start the experiment and from genotypes taken from multiple time points over the course of an adaptive walk in two evolved lineages that differed in the number of mutations fixed and final mean fitness (see Materials and Methods). Multiple beneficial mutations often arise independently as sectors in different locales during a single growth cycle (Figure 1B). Thus, sampling a colony from predetermined positions at the same radius on a plate and assaying fitness of these colony isolates allows us to estimate the distribution of fitness effects among mutations that arise and escape initial stochastic loss but have yet to fix.

**Figure 5:**
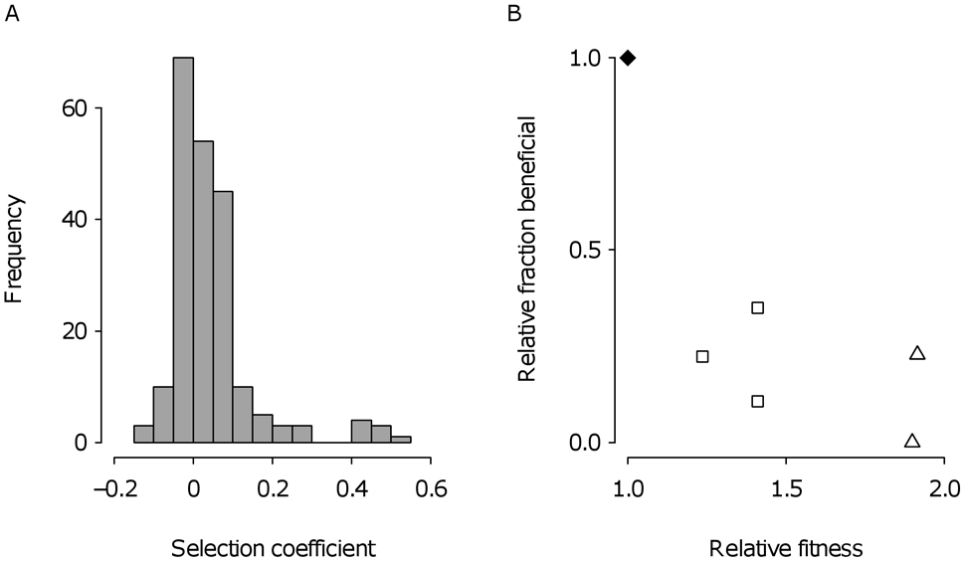
Mutations available to selection. (A) Distribution of selection coefficients among 210 isolates, each sampled from a predetermined position of a 5-d-old colony of the genotype used to found the selection experiment. (B) The relationship between the fraction of isolates that are beneficial and mean fitness of the genotype from which they were derived in two lineages from the large bottleneck treatment (scaled to the ancestor and corrected for false positives, see Materials and Methods and Figure S3). The fraction of isolates that are beneficial is reported relative to the fraction at the starting of the experiment. Symbols used correspond to those shown in Figure 2A. Fewer mutations were available to the final evolved types than the ancestor (Welch's two-sample *t*-test on proportions assuming unequal variance, low-fitness lineage: *t*_213.76_ = −11.27, *p*<0.0001; high-fitness lineage: *t*_284.72_ = −4.99, *p*<0.0001).

A representative distribution of fitness effects among 210 mutants collected from the founding genotype is shown in Figure 5A. The distribution is modal near zero with a substantially smaller mean (±s.d.) among beneficial mutations (0.13±0.076) than for the first mutation fixed in both treatments (large bottleneck: 0.21±0.085; *t*-test assuming unequal variance, *t*_105.2_ = 6.23, *p*<0.0001; small bottleneck: 0.20±0.16, *t*_73.8_ = 3.28, *p* = 0.0016) and a long right tail representing putatively accessible beneficial mutations. More striking is the relationship between mean fitness of the evolving population and the fraction of beneficial mutations available to selection, which is negative (Figure 5B) even after accounting for multiple comparisons (Materials and Methods and Figure S3). This result implies that the supply of beneficial mutations is depleted as a population adapts in both lineages.

## Discussion

We have experimentally studied properties of adaptive evolution in mutation-limited populations by measuring fitness through evolutionary time. Previous work has provided insight into the properties of the first step of an adaptive walk, from the distribution of mutations prior to selection to those fixed [2],[4],[7],[9],[44]. Here, we have focused on the properties of multiple steps in an adaptive walk by monitoring 118 replicate evolving populations and inferring the number and the fitness effects of new mutations in each lineage. Our main findings can be summarized as follows. First, the distribution of fitness effects among mutations fixed by selection remains approximately bell-shaped at each step in an adaptive walk (Figure 3). Second, the adaptive walks we have studied (specifically, those involved in the recovery of fitness due to a costly resistance mutation) tend to be short, ranging between one and three steps (Figure 4). Third, both the supply of beneficial mutations and the fitness gains associated with each step of an adaptive walk decline as mean fitness increases (Figure 5). Taken together, these results imply that the gradualist view of evolution is incorrect; rather, the bulk of adaptation in mutation-limited populations is likely to be achieved by the first few mutational steps.

Interestingly, our conclusions are not much altered by the bottleneck size used between serial transfers. This result is attributable to the opposing effects of drift and clonal competition in small and large populations. In small populations, only mutations of moderate to large effect are likely to escape stochastic loss when rare, and those that do are nearly guaranteed to fix due to the absence of clonal competition. In large populations, more mutations of small effect will reach appreciable frequencies but are eventually lost due to clonal competition. The result is that the mean fitness effect of mutations that fix under different population sizes remains approximately the same at each step throughout the course of an adaptive walk. By contrast, population size does affect the mean fitness among lineages achieved by the end of the experiment, with small populations having lower fitness than large populations by the end of the experiment. This observation is consistent with our finding of fewer fixed mutations in small compared to large populations, which likely stems from changes in the mutation supply rate, *Nμ* (where *N* is population size and *μ* mutation rate). Importantly, even in our small bottleneck treatment, *N* is unlikely to be so low as to prevent sampling of a large number of single-step mutations, as required by theory [14],[41].

As with most selection experiments, we have little direct information about the topography of the adaptive landscape; however, two results suggest that it may be fairly rugged. First, fitness tends to plateau at different levels in most lineages by about generation 500, and these fitness differences are maintained throughout the experiment [48]. Second, the brevity of the adaptive walks observed here is consistent with that expected under a rugged landscape; smoother landscapes containing fewer, but higher, fitness peaks lead to longer adaptive walks [15].

The shortness of the adaptive walks is a striking feature of our results that stands in contrast to modestly longer walks of three to six steps documented in bacteria [28],[31], yeast [27],[30], a virus [32], and a single example of an extremely long walk of 14 steps in a virus [29]. Our result is likely to be robust because it is based on statistical inferences that have been independently validated through experiment and that come from the analysis of between one and two orders of magnitude more populations than previous experiments. Nevertheless, the generality of our results may be questioned on two grounds. First, adaptive walks may appear short if selection is negatively frequency-dependent, as can often arise in spatially structured environments [39],[49],[50]. As we have pointed out, this explanation seems unlikely because our transfer procedure destroyed any spatial structure that may have arisen during colony expansion, and there were no obvious indications that distinct morphotypes coexisted for multiple transfers. Thus, although we cannot exclude the possibility that frequency-dependent selection may have gone undetected in some lineages, we have no compelling reason to suspect that it is widespread in our experiment.

Second, it may be argued that short adaptive walks are expected a priori because we are studying compensatory evolution, that is, the recovery of fitness due to the presence of a costly resistance mutation [51],[52]. However, there seems little reason to believe that compensatory evolution should differ qualitatively from more “open-ended” instances of adaptive evolution. All adaptive evolution is compensatory to a degree, in the sense that wild-type fitness declines initially either due to the fixation of a costly mutation, as in classic compensatory evolution, or due to a change in the environment. Indeed, this view is central to mutational landscape models [8],[27] that see adaptation being initiated by a change in environment that causes a drop in the rank of wild-type fitness: the lower the fitness rank of the wild type, the larger the variety of mutational routes to adaptation available. Although our experiment did not test this prediction directly, we do know that a wide range of mutational routes to adaptation were taken by our evolved lineages, as evidenced by the observation of very few back mutations restoring sensitivity to fungicide (just five out of 118 lineages) and substantial genotype-by-environment interaction for fitness among all evolved populations. Furthermore, several evolved populations achieved a significantly higher fitness by the end of the experiment than the fungicide-sensitive ancestral strain (see, for example, Figure S1), suggesting that compensatory evolution need not constrain the founding population to exploring a single fitness peak. We thus expect our results to apply with equal force to any situation where the drop in fitness of our founding strain is of comparable magnitude.

Why did we observe shorter adaptive walks than previous experiments? One possibility is that our founding strain was already well adapted to the conditions of growth and so had only a few beneficial mutations available to it. This explanation is difficult to reconcile, however, with the observation of substantial genotype-by-environment interaction mentioned above, the apparently large fraction of beneficial mutations available to the ancestor (Figure 5A) and a gain in mean fitness of approximately 48% in the large bottleneck treatment. Notably, such a fitness increase is comparable in magnitude to that observed in experiments with yeast [27],[30] and exceeds that observed in much longer-duration experiments with bacteria [28],[31]. (Note that low ancestral fitness is likely the explanation for the long walk observed in the viral experiment [29], where the fitness of the evolved lineages increased by approximately 364% over the ancestor). A second possibility is that we did not run our experiment long enough, and adaptive walks monitored here did not yet reach a peak. However, the observation that the vast majority of populations have reached a fitness plateau by 500 generations strongly suggests we have captured the bulk of adaptation, and the few additional adaptive mutations that could occur if we ran the experiment longer would not qualitatively alter our conclusions. Finally, the observation that adaptive evolution in most lineages ceases by about generation 500 suggests that the underlying adaptive landscape remains relatively constant in our experiments. It is notable that, in at least two experiments where longer walks have been noted, adaptive landscapes have been observed to change over the course of the experiment, as evidenced by declines in mean fitness, relative to the ancestor, in evolved populations [27] and the emergence of negative frequency-dependent selection [53].

That the relationship between the mean fitness increases with each successive step in an adaptive walk is negative suggests two important insights into the mechanics of adaptive evolution. First, the distribution of fitness effects among beneficial mutations prior to selection is expected to lie in the Weibull domain of attraction [17], a decreasing distribution with a truncated right-hand tail. In other words, there are more small-effect than large-effect mutations, and there is an upper limit to mutation size. It is notable that the prediction of a right-truncated distribution of fitness effects among beneficial mutations is one possible outcome of heuristic models based on EVT as well as the biologically more realistic Fisher's geometric model [21]. Second, adaptive evolution in our experimental system is best viewed as involving the successive substitution of single mutations. This conclusion derives from the fact that the relationship between mean fitness increase and number of steps remains negative regardless of bottleneck size, a result that can only occur if multiple co-occurring mutations make little or no contribution to the fitness increases associated with adaptation.

Our results have two important practical implications. First, if the environment changes regularly, populations may be expected to adapt perpetually, and it will be difficult to make strong predictions as to the outcome, for example, of long-term changes to the environment such as those expected to occur under many climate change scenarios. Second, compensatory evolution in response to losses in fitness due to mutations conferring resistance to fungicides, antibiotics, or other chemotherapies, is likely to be fast, requiring only a few mutational steps. More fundamentally, the picture emerging from these experiments is that adaptive evolution in mutation-limited populations can be readily understood from rather simple models that, because they are largely based on the statistical properties of extreme events, are likely to be robust to biological details.

## Materials and Methods

### Strains and Growth Conditions

We used *A. nidulans* strain WG615 (*w*A3, *fld*A1, *pyro*A4, *ve*A1) for the selection experiment, kindly provided by Fons Debets and Marijke Slakhorst at Wageningen University, the Netherlands. This strain is resistant to the fungicide fludioxonil (*fld*A1). Resistance confers a cost of around 46% relative to the sensitive strain from which it was derived when growing in the absence of fungicide [33]. Colony diameter of WG615 after 5 d of growth at 37°C (MGR) was 41.0 mm (s.d.: 2.60). For comparisons with a fungicide-sensitive strain, we used strain WG638 (*y*A1, *ve*A1), which has the same genetic background as WG615. In the selection experiment, spores and mycelium were washed from the plate using 5 ml of saline-Tween (water containing NaCl 0.8% and Tween-80 0.05%), reserving 5 µl for transfer to fresh medium and storing 0.8 ml of the mixture mixed with 0.3 ml 80% glycerol at −80°C. Bottleneck sizes were adjusted by dilution. For all experiments, we used solid Complete Medium (CM) set at pH 5.8, consisting of NaNO_3_ 6.0 g/l; KH_2_PO_4_ 1.5 g/l; MgSO_4_.7H_2_O 0.5 g/l; NaCl 0.5 g/l; 0.1 ml of a saturated trace element solution containing FeSO_4_, ZnSO_4_, MnCl_2_, and CuSO_4_; tryptone 10 g/l; and yeast extract 5 g/l and (added after autoclaving) glucose 4.0 g/l. Cultures were incubated at 37°C.

### Selection Experiment

We initiated 120 replicate populations from *A. nidulans* strain WG615 by placing 5 µl of a dense spore suspension (>10,000 spores) in the center of a Petri dish containing solid CM medium. We propagated these replicate lineages by serial transfer for a total of 800 generations. Every 5 d, (∼80 mitotic generations [33],[34]), we transferred a 5-µl random sample of a mature colony containing either 50,000 or 500 nuclei to fresh medium (large and small bottlenecks, respectively) obtained from a final population size of approximately 10^9^ nuclei. An aliquot of the transferred sample was stored at −80°C. Our bottlenecking scheme ensures that effective population size in our treatments differs by at least two orders of magnitude. Two evolving lineages from the large bottleneck treatment were lost due to infection. Note that the number of generations elapsed between transfers depends primarily on cell-cycle duration rather than bottleneck size because our populations expand radially at a constant rate and so lack a stationary phase [33].

### Fitness Assays

We measured fitness of the *i*th genotype (*w_i_*) as the colony diameter (mycelial growth rate; MGR) after 5 d of incubation, which is widely used as a measure of fitness and is highly correlated with other fitness measures such as total spore production and biomass [33],[54],[55], as well as competitive fitness (based on competitions between seven strains displaying substantial variation in MGR and a genetically marked ancestor, see Text S4: *r* = 0.81, *t*_5_ = 3.09, *p* = 0.027). Selection coefficients were calculated as *s* = (*w_i_*−*w*_ancestor_)/*w*_ancestor_. Each lineage was assayed in triplicate at multiple time points (0, 80, 160, 240, 320, 480, 640, and 800 generations) in a single assay. Two lineages from the large bottleneck treatment were eliminated from the maximum likelihood analysis due to missing data. For 26 evolved strains that had a high relative fitness, we reassayed the fitness at 800 generations using at least seven replicates together with the fungicide-sensitive strain WG638. We estimated fitness of all lineages at the end of the selection experiment in CM supplemented with 0, 0.05, 0.2, or 0.4 ppm of fludioxonil in duplicate and used an analysis of covariance to test the main effect of lineage (genetic variance) and the interaction between lineage and fludioxonil concentration (genotype-by-environment interaction variance).

### Sexual Crosses

We performed sexual crosses [42] to assess the number of loci fixed by crossing evolved genotypes derived from WG615 with strain WG561 (*fld*A1, *lys*B5, *ve*A1). We estimated the fitness of at least 50 progeny for each of 15 crosses and used the Castle-Wright estimator [35] modified for haploids [30] (see Table S1 and Text S3). Strain WG638, which has high fitness, was included in all fitness assays as a reference strain.

### Mutations Available to Selection

We initiated replicate populations of genotypes of interest by placing 5 µl of a dense spore suspension in the center of a Petri dish. After 5 d of colony expansion, we sampled approximately 3 mm^2^ of mycelium including spores at three preassigned locations on the edge of the colony. Fitness was estimated for each spore sample, and the fraction of samples with fitness greater than that of the parent genotype was calculated for each time point. We assayed fitness in blocks and used strains WG615 and WG638 as reference strains to account for variation between blocks. Logistic regressions were performed in *R* version 2.6.1 using generalized linear models with binomial errors. We controlled for false positives, which would inflate our estimates of the fraction of beneficial mutations, using a false discovery rate analysis [56]. Using a range of false discovery rates reduces the number of mutations identified as beneficial in all lineages (see Figure S3), but does not qualitatively change our results.

## Supporting Information

Figure S1 **Fitness of 28 evolved lineages after 800 generations (grey bars), the ancestor that founded the selection experiment, and a strain that is fungicide sensitive but has the same genetic background.** Error bars show 95% confidence intervals. The founding strain of the evolved lineages carries a fungicide resistance mutation that is costly under the growth condition used here (Schoustra et al., 2006 [33]). Fifteen of these lineages have a fitness that is significantly different (higher or lower) than the fungicide-sensitive strain with the same genetic background. A post hoc Tukey test after an ANOVA (*F*_29,243_ = 70.0, *p*<0.0001) revealed that at least three strains have significantly higher fitness than the sensitive strain. Figure 1C in the main text shows one of the evolved lineages with higher fitness than the sensitive ancestor; other than its colony diameter, which is 6 mm larger than the fungicide sensitive strain, the evolved strain has the same physical appearance as the fungicide sensitive strain.
(0.56 MB TIF)

Figure S2 **Relationship between the number of nuclei present in a mycelium (ln transformed number of colony forming units [CFU]) as a function of the size of the fungal colony (colony diameter in millimeters).**
(0.00 MB EPS)

Figure S3 **Mutations available to selection.** Fraction of mutations sampled available to selection, scaled to the fraction available to the ancestor. (See also Figure 5B in main text.) Data were adjusted by false discovery rate (FDR) control (Verhoeven et al., 2005 [56]) to remove putatively spurious beneficial mutations. Results using multiple FDR cutoffs are presented (listed above each plot). The rate used in the Figure 5B presented in the main text is 0.15. Our qualitative conclusions do not change for a very large range of false discovery thresholds.
(0.01 MB EPS)

Figure S4 **Average time to quasifixation of beneficial mutations as a function of their selection coefficient and the size of the population (see Text S1).** Dots denote the average of simulations tracking the time to fixation or loss of 10,000 new mutations with selection coefficient *s*. Continuous lines are a diffusion-based approximation for the average time to quasifixation, assuming the strong selection limit (specifically, **T**_fixation_ = 2 ln[*N_e_s*]/*s*).
(0.02 MB EPS)

Table S1 **Comparison of number of loci contributing to adaptation estimated with Caste-Wright estimator and maximum likelihood.** Number of segregating loci (*n_e_*) in a cross between evolved genotypes and the non-evolved ancestor, using the Castle-Wright estimator [30] (with and without the correction suggested by [57]) and the number of mutations fixed estimated by the maximum likelihood (ML) program.
(0.04 MB DOC)

Text S1 **Likelihood framework for estimating selection coefficients of beneficial mutations spreading in each population from fitness trajectory data.**
(0.06 MB DOC)

Text S2 **Experimental evidence for exponential population growth.**
(0.03 MB DOC)

Text S3 **Estimation of the number of segregating loci using the Castle-Wright estimator.**
(0.03 MB DOC)

Text S4 **Comparison between mycelial growth rate and competitive fitness.**
(0.03 MB DOC)

## Abbreviations

EVT: extreme value theory
ML: maximum likelihood
