# Supplementary figures and images for: The Properties of Adaptive Walks in Evolving Populations of Fungus
Source: PLoS Biol. 2009 Nov 24;7(11):e1000250. doi: 10.1371/journal.pbio.1000250 (PMC2772970; doi:10.1371/journal.pbio.1000250)

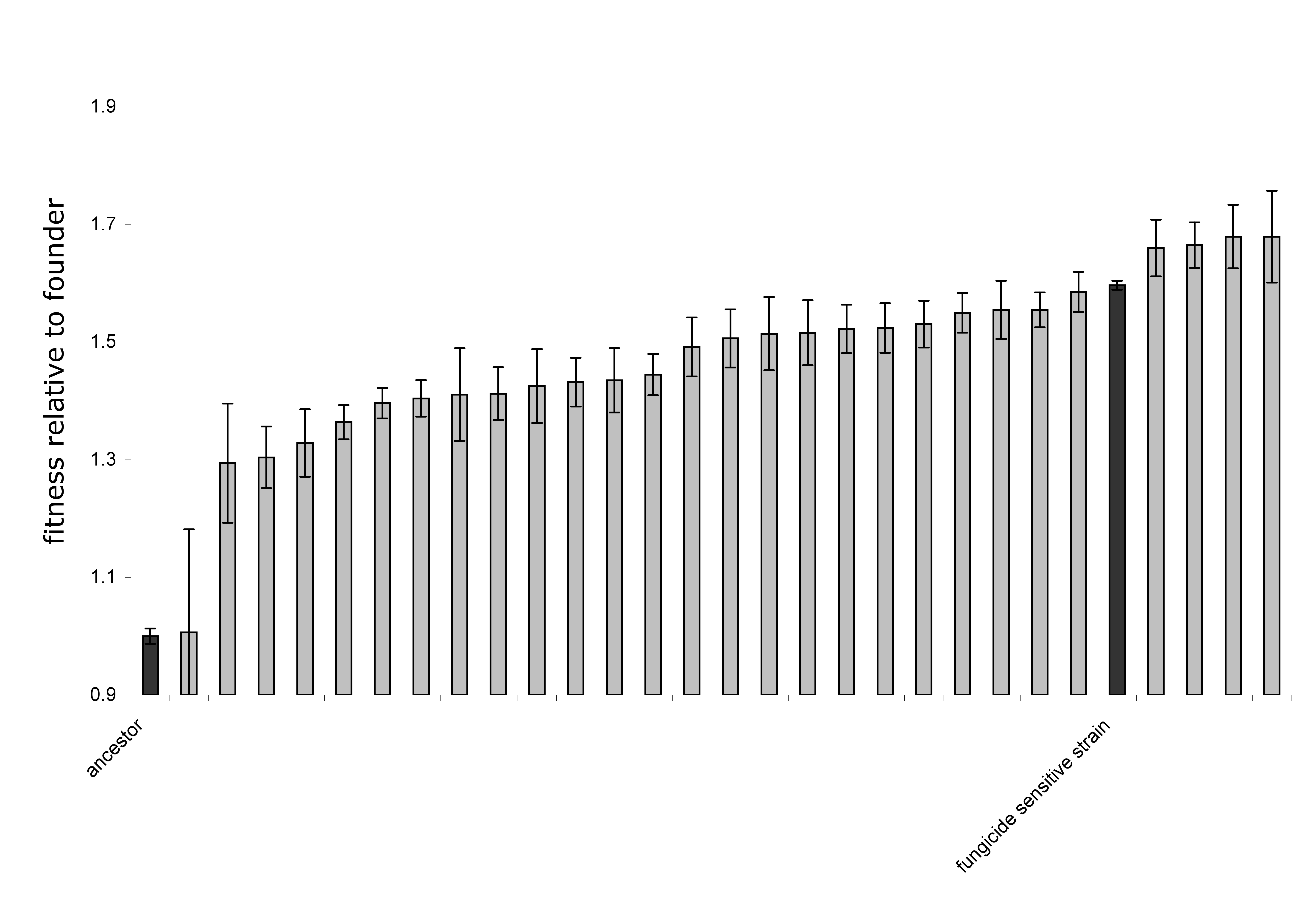

Supplement: Figure S1 — Fitness of 28 evolved lineages after 800 generations (grey bars), the ancestor that founded the selection experiment, and a strain that is fungicide sensitive but has the same genetic background. Error bars show 95% confidence intervals. The founding strain of the evolved lineages carries a fungicide resistance mutation that is costly under the growth condition used here (Schoustra et al., 2006 [33]). Fifteen of these lineages have a fitness that is significantly different (higher or lower) than the fungicide-sensitive strain with the same genetic background. A post hoc Tukey test after an ANOVA (F 29,243 = 70.0, p<0.0001) revealed that at least three strains have significantly higher fitness than the sensitive strain. Figure 1C in the main text shows one of the evolved lineages with higher fitness than the sensitive ancestor; other than its colony diameter, which is 6 mm larger than the fungicide sensitive strain, the evolved strain has the same physical appearance as the fungicide sensitive strain. (0.56 MB TIF) [file pbio.1000250.s001.tif]
