# Supplementary material for: The Properties of Adaptive Walks in Evolving Populations of Fungus
Source: PLoS Biol. 2009 Nov 24;7(11):e1000250. doi: 10.1371/journal.pbio.1000250 (PMC2772970; doi:10.1371/journal.pbio.1000250)
Supplement: Table S1 — Comparison of number of loci contributing to adaptation estimated with Caste-Wright estimator and maximum likelihood. Number of segregating loci (ne) in a cross between evolved genotypes and the non-evolved ancestor, using the Castle-Wright estimator [30] (with and without the correction suggested by [57]) and the number of mutations fixed estimated by the maximum likelihood (ML) program. (0.04 MB DOC) [file pbio.1000250.s005.doc]

Supplementary Table 1. Number of segregating loci (*n_e_*) in a cross between evolved genotypes and the non-evolved ancestor, using the CW estimator (with and without the correction suggested by [Zeng 1992]) and the number of mutations fixed estimated by the maximum likelihood (ML) program.

| Cross | *n_e_* based on CW estimator | *n_e_* based on CW with correction | # fixations estimated by ML program |
| --- | --- | --- | --- |
| A | 0.42 | -0.16 | 1 |
| B | 2.7 | 4.42 | 2 |
| C | 2.46 | 3.92 | 2 |
| D | 1.75 | 2.5 | 2 |
| E | 2.2 | 3.4 | 2 |
| F | 0.78 | 0.57 | 1 |
| G | 1.18 | 1.37 | 1 |
| H | 1.29 | 1.58 | 2 |
| I | 1.7 | 2.39 | 3 |
| J | 1.72 | 2.39 | 3 |
| K | 0.73 | 0.46 | 1 |
| L | 0.95 | 0.9 | 1 |
| M | 0.76 | 0.5 | 2 |
| N | 3.09 | 5.18 | 2 |
| O | 2.46 | 3.92 | 2 |
