# Supplementary material for: The Properties of Adaptive Walks in Evolving Populations of Fungus
Source: PLoS Biol. 2009 Nov 24;7(11):e1000250. doi: 10.1371/journal.pbio.1000250 (PMC2772970; doi:10.1371/journal.pbio.1000250)
Supplement: Text S1 — Likelihood framework for estimating selection coefficients of beneficial mutations spreading in each population from fitness trajectory data. (0.06 MB DOC) [file pbio.1000250.s006.doc]

**Text S1. Likelihood framework for estimating selection coefficients of beneficial mutations spreading in each population from fitness trajectory data**

**Assumptions of the model.** We model a population composed of organisms that are strictly clonal. All clones in the population grow at exponential rates that do not vary in time or depend on the frequencies of other clones in the culture. We further assume that during propagation clones are serially transferred to fresh media. Between each transfer episode there is no density dependence. We treat the problem of bottlenecks deterministically by assuming that the minimum population size during each bottleneck is large enough to not affect the trajectories of allele frequencies between bottlenecks. Incorporating drift would in theory be feasible, for instance by using a backward diffusion equation and integrating over several possible paths of trajectories conditioning on escaping initial stochastic loss. But this would require developing a Monte Carlo approach involving computational challenges that are beyond the scope of this study (see Bollback et al 2008 for a solution when only a single mutation is considered at a time). In our case neglecting drift seems fair since the smallest bottleneck used in our experiment is around 500 individuals, although we acknowledge that the actual frequency trajectories of clones (conditional on escaping stochastic loss) may be somewhat steeper than the deterministic expectation we use. Note also that these assumptions mean that our procedure makes inferences about beneficial mutations that escape stochastic loss and reach appreciable frequency; those lost to drift are not detected.

**Derivation of the likelihood function.** In general we wish to estimate a vector of Malthusian parameters r = (*r*_1_, *r*_2_, … *r_n_*) and times of origin t = (*t*_1_, *t*_2_, … *t_n_*) of up to *n* clones (*n* -1 beneficial mutations and the ancestral clone) from data consisting of fitness estimates (observations) collected at *k* spaced time points during the adaptation of a single population.

The likelihood of the data $D=\left(w_{1},w_{2},...,w_{k}\right)$ consisting of fitness estimates at *k* time points is then

$L(D|r,t)=\prod_{i=1}^{k} E\left[prob(w_{i },T_{i})\right]$, (1)

where *E*[ . ] is the expectation over all possible clones present at each time point in the population assayed and $prob(w_{i },T_{i})$ denotes the probability of observing a fitness *w_i_* at time point *T_i_* .

Writing out the expectation by conditioning on which clone (out of *n* possible clones) is picked up at time *T_i_* yields:

, (2)

where $\bar{p}_{j}$(*T_i_*) is the expected frequency of clone *j* at time *T_i_* and$f_{e}(r_{j},w_{i })$the probability of observing fitness estimate *w_i_* at time point *T_i_* given that clone *j* was picked at that time point. Note that all we need for calculation of the likelihood is the (unconditional) expected frequency of clones (see below for recursions used). That is, we do not need information about whether or not the clone spreads to fixation, higher moments of the distribution of offspring number, or other complications. The likelihood does, however, depend on the other values of *r* and *t* thus allowing their estimation from the pattern of temporal increase in fitness.

To calculate $\bar{p}_{j}$(*T*) the expected frequency of clone *j* at time *T*, begin with *N_j_* (*T*), the number of individuals of that genotype at time of observation *T*:

$\bar{p}_{j}\left(T\right)=E\left[\frac{N_{j}\left(T\right)}{N\left(T\right)}\right]\approx\frac{\bar{N}_{j}\left(T\right)}{\bar{N}\left(T\right)}.$ (3)

The approximation is justified because $N=\sum_{j} N_{j}$ is always so large in our experiments (at least 10^9^ nuclei) that it can be treated deterministically. In practice, the bookkeeping necessary to calculate each clone census size at each time point (*N_j_*(T)’s) is implemented as a set of recursions (see below).

Population growth is modelled as cycles of exponential growth in continuous time followed by the transfer of n* individuals to fresh media. *N_j_*(T), the number of individual from clone *j* at time T, is calculated as :

*N_j_*(T) = 0 if T< *t*_j_ (the clone *j* has not appeared yet),

*N_j_*(T) = exp(r_j_(T- *t*_j_)) if the clone *j* appeared during that cycle,

*N_j_*(T) = n* *ñ_j_(t) /* exp(r_j_T*) if clone *j* appeared during a previous cycle.

Here, Δ is the length of time between transfers (and bottleneck) and *T** is the amount of time since the last transfer and n* the number of individual transferred at each cycle. The quantity *ñ_j_*(*t*) is the number of individuals of genotype *j* just before the last transfer. In the second of the three cases outlined above, genotype *j* has experienced uninterrupted exponential growth for T-*t*_j_ since it appeared in a single individual within a current cycle. In the last case, the genotype *j* started the current cycle at a frequency *ñ_j_(t) /*, and the number of individuals of this genotype at the start of the cycle is that frequency times n*. This number is then multiplied by their growth factor, which is exp{*r_j_*T*}.

We maximize this likelihood with respect to *r* and *t*. We are not necessarily interested in the vector of times of origin of clones, *t*, so they act here as nuisance parameters. An alternative approach would be to assume a model for the appearance of the mutations, so that these times need not be estimated. There are two reasons why this latter approach is not attractive. First, it would require that we estimate yet other parameters (the mutation rates, in particular), and so just transfer the problem to another place. Second, the assumptions needed are not appealing. For example, we would need to assume something like constant probabilities of appearance of a new clone per replication. But we expect that the origin of some genotypes requires previous mutations in the genetic background, so these probabilities are not expected to be constant. In short, treating the times of origin *t* as parameters to be estimated seems so far like the best approach.

To convert colony expansion of fungal mycelium (mycelial growth rate, MGR) to exponential growth rates in terms of the number of individual nuclei present in the population (*w*’s, see Methods Summary) we used the following transformation: *w* = 0.0437*MGR/10. This transformation comes from fitting an exponential model between CFU and MGR of the form, CFU = A*exp^b*mgr^ and estimating *b*. Division by 10 is simply a scaling factor used here for numerical convenience and avoiding overflow when calculating clone census size.

**Choosing an error function *f*_e_.** A Gaussian distribution of the errors with variance σ^2^ around the fitness estimates was used:

*f*_e_ (*r*_j_,*w*_i_)=.

This choice is likely a robust one because we work with mean estimates averaged over several independent replicates (due to the central limit theorem, these estimates will be quite close to Gaussian). This choice is very flexible and alternative error functions can be chosen if the data strongly suggests that one should do so.

**Model selection.** The likelihood calculations and maximization is implemented as an ANSI C-program (available upon request) that takes as input the list of fitness observations at known time intervals. The user provides estimated standard errors around the fitness estimates at each time point. The program fits sequentially a model with 1, 2, 3, etc clones to a given observed fitness trajectory from the selection experiment. Note that the model does not assume any nesting relationship between clones, meaning that clone *i*+1 does not necessarily arise in the genetic background of clone *i*. We use Akaike’s information criterion (AIC) for model selection and deciding how many clones provide the best fit to the data collected in each population. The AIC criterion does not ensure that each clone in the selected model has necessarily reached complete fixation. For example, those populations that are best fitted with a 4-clone model (meaning 3 beneficial mutations), the last mutation may be still on its way to fixation and some earlier clones may have been displaced before reaching fixation.

We did two things to ensure that the ML results provided reasonably accurate interpretations of the number and fitness effect of mutations fixed. First, we used Monte Carlo simulation to check that the timescale of our experiment (>800 generations) and the effective population sizes used were compatible with the time to reach quasi-fixation for the range of selection coefficients inferred. These simulations were run assuming a Wright-Fisher model of haploid selection with a range of selection coefficients (*s* > 0.02). We defined quasi-fixation as the time to rise in frequency from 1/*N_e_* to 1-1/(*N_e_ s*), which is reasonable because mutations always introduces new genotypes such that strict fixation (frequency = 1) is probably never achieved in real populations. Moreover a quasi-fixation frequency of 1-1/(*N_e_ s*) is very close to 1 as *Ne s* grows and corresponds to the frequency threshold where the beneficial mutation being considered is so common that drift overwhelms natural selection in determining true fixation (Gale 1990, pp261-265). Taking account of the bottlenecking scheme used in our experiment, we assumed *N_e_*= 1500 for the small bottleneck treatment and *N_e_* =150,000 for the large bottleneck treatment. For each combination of *N_e_* and *s* we monitored the time to either loss or quasi-fixation of 10000 new beneficial mutations appearing initially as single mutants. These simulations (see Figure S4) show that, over the duration of our experiment, the majority of mutations have enough time to reach quasi-fixation in both bottleneck treatments.

Second, we checked that the ML procedure was not grossly underestimating the number of mutations fixed in our population through direct estimates of the number of segregating mutations resulting from crosses between the ancestral genotype and derived populations (see below). As mentioned in the text, the correspondence between the number of beneficial mutations inferred from the ML procedure and our experimental estimates derived from sexual crosses was very good.

**Implementation of the likelihood.** For each model the likelihood is maximized using the Simplex algorithm as implemented in the routine *amoeba* (Press et al. 1992). The programs outputs estimate the MLE of *r*’s and *t*’s as well as the likelihood of the data under each model. If the ML program finds that a model with multiple clones gives a better fit than a fit with just one clone, this is an indication that a beneficial mutation has fixed.

**References**

Bollback, JP , York, TL, Nielsen, R. (2008) Estimation of 2N_e_s from temporal allele frequency data. Genetics 179, 497–502.

Gale, J.S. (1990) *Theoretical Population Genetics.* Uwin Hyman Ltd, London

Press, W.H., Flannery, B.P., Teukolsky, S.A., Vetterling, W.T. (1992) *Numerical Recipes in C: The Art of Scientific Computing*. Cambridge University Press.
