# Supplementary material for: The Properties of Adaptive Walks in Evolving Populations of Fungus
Source: PLoS Biol. 2009 Nov 24;7(11):e1000250. doi: 10.1371/journal.pbio.1000250 (PMC2772970; doi:10.1371/journal.pbio.1000250)
Supplement: Text S2 — Experimental evidence for exponential population growth. (0.03 MB DOC) [file pbio.1000250.s007.doc]

**Text S2. Experimental evidence for exponential population growth.**

We verified that an exponential model provides an adequate description of population growth between transfers by inoculating 5 µl of spores of strain WG638 in the center of CM Petri dishes and allowing the colonies to expand (at 37 ^o^C) for different periods of time (40, 47, 62, 69, 96, 112, 115 and 120 hours). Four replicate plates were inoculated for each time point and both MGR and the number of colony forming units (CFU) were assayed for all time points simultaneously (meaning that inoculation was staggered through time such that all plates could be harvested on the same day). CFU was estimated as the number of viable nuclei on solid medium after washing both spores and mycelium off a plate using 10 ml of saline-Tween (water with 0.08% NaCl and 0.005% tween-80), vortexing vigorously for 45 seconds, and then plating an appropriate dilution. Under exponential growth the relationship between ln(CFU) and MGR will be linear. Our results, shown in Figure S2, are consistent with this prediction. A linear regression gives a slope of 0.0437 with a 95% confidence interval of 0.01 and an adjusted R^2^ of 0.87. Thus an exponential growth model provides an adequate description of our data.
