# Supplementary material for: The Properties of Adaptive Walks in Evolving Populations of Fungus
Source: PLoS Biol. 2009 Nov 24;7(11):e1000250. doi: 10.1371/journal.pbio.1000250 (PMC2772970; doi:10.1371/journal.pbio.1000250)
Supplement: Text S3 — Estimation of the number of segregating loci using the Castle-Wright estimator. (0.03 MB DOC) [file pbio.1000250.s008.doc]

**Text S3. Estimation of the number of segregating loci using the Castle-Wright estimator**

We verified the results of the ML procedure outlined above by performing crosses between evolved genotypes and the ancestral genotype (WG561) and using the variance in fitness among the F1 progeny and the two parental genotypes to estimate the effective number of segregating loci, *n_e_*, using the Castle-Wright (CW) estimator (Castle 1921) modified for haploids (as in Zeyl 2005): *n_e_ =* [ (­*z*_P_*_1_* – *z*_P_*_2_* )^2^ – var(*z*_P_*_1_*) – var(*z*_P_*_2_*) ] / 4 var(F1), where *z*_P_*_i_* is the mean fitness of the parental types and var(F1) is the variance in fitness among the F1 offspring. Crosses were performed following the procedure of Clutterbuck (1974). Measures of fitness (mycelial growth rate) of at least 40 progeny from each of 15 crosses were used to estimate the number of segregating loci. Table S1 shows our results, along with the corresponding number of mutations inferred from the ML program. The methods give comparable results (paired *t*-test: *t*_14_ = -0.99, *P* = 0.34). Zeng (1992) has suggested a correction to account for the possibility of unequal fitness effects at different loci and linkage (also see Zeyl 2005). We applied Zeng’s (1992) correction for unequal fitness effects, assuming an exponential distribution (CV = 1). We did not correct for linkage since we have no a priori reason to suspect tight linkage (recombination rate = 0.5) Applying the correction did not result in qualitatively different results (Table S1; paired *t*-test: *t*_14_ = 1.16, *P* = 0.27).

It is well known that the CW estimator may underestimate the true number of segregating loci, even after making the corrections by Zeng (1992), for two reasons. First, the segregational variance attributable to any given site becomes small and difficult to estimate accurately when the number of loci is large (large here means above 10 loci; Lande 1981, Lynch and Walsh 1998). Secondly, the maximum number of estimated loci is given by the haploid number of chromosomes multiplied by the number of recombinational events. Therefore we urge caution in interpreting the results reported in Table S1. Nevertheless three lines of evidence suggest our results are not likely to be severely biased. First, the number of mutations we estimated is substantially less than 8, the number of linkage groups (an estimate of the number of chromosomes) in *A. nidulans*. Given that *A. nidulans* is also known to have a high meiotic recombination rate (Clutterbuck 1997), our CW estimates are unlikely to have been limited by the extent of recombination. Second, extensive simulation studies by Otto and Jones (2000) have shown that when the true number of loci is on the order of 2-10 Zeng’s corrected CW estimator does not systematically underestimate the true number of loci. Third, our own simulations of the time to quasi-fixation for beneficial mutations (see Fig. S4) suggest that, given the range of fitness values we observed, no more than 3-4, and at most 5, mutations could have been substituted over the approximately 800 generations of our experiment. Thus, our estimates of between 1 and, at most 5, loci are unlikely to have severely underestimated the true number of beneficial mutations substituted.

**References**

Castle, W.E. (1921) An improved method of estimating the number of genetic factors concerned in cases of blending inheritance. Science 54, 223 (1921).

Clutterbuck A.J. (1974) *Aspergillus nidulans*. Pp 447-510 *in* P.C. King Ed. *Handbook of Genetics*. Plenum Press, New York.

Clutterbuck A.J. (1997) The validity of the *Aspergillus nidulans* linkage map. Fungal Genetics and Biology 21, 267-277.

Lande, R (1981) The minimum number of genes contributing to quantitative variation between and within populations. Genetics 99, 541-553.

Lynch M, Walsh B (1998) Genetics and Analysis of Quantitative Traits. Sunderland: Sinauer Associates, Inc.

Otto S.P., Jones C.D. (2000) Detecting the undetected: estimating the total number of loci underlying a quantitative trait. Genetics 156: 2093-2107.

Zeng, Z-B. (1992) Correcting the bias of Wrights estimates of the number of genes affecting a quantitative character: a further improved method. Genetics 131**,** 987-1001.

Zeyl, C. (2005) The number of mutations selected during adaptation in a laboratory population of *Saccharomyces cerevisiae*. Genetics 169**,** 1825-1831.
