# Supplementary material for: The Properties of Adaptive Walks in Evolving Populations of Fungus
Source: PLoS Biol. 2009 Nov 24;7(11):e1000250. doi: 10.1371/journal.pbio.1000250 (PMC2772970; doi:10.1371/journal.pbio.1000250)
Supplement: Text S4 — Comparison between mycelial growth rate and competitive fitness. (0.03 MB DOC) [file pbio.1000250.s009.doc]

**Text S4. Comparison between mycelial growth rate and competitive fitness.**

Seven strains were chosen from the selection experiment and competed against a common (fungicide sensitive) tester strain WG562 (*lys*B5). Competitions were performed in triplicate by mixing spores of the selected strain with spores of the tester strain. The ratio of the spores of the two strains was estimated using serial dilutions and plate counts and 5 µl of the spore mixture was inoculated in the center of a Petri dish containing solid CM medium. After 5 days (80 generations) of incubation, all spores were washed off the surface of the colony and a 5 µl random sample was transferred to fresh medium. After another 5 days of incubation all spores were washed off and the ratio of spores formed by the two strains was estimated. The selection coefficient was calculated as *s* = [ln (ratio in final mixture) - ln (ratio in initial mixture)]/160 generations. Mycelial growth rate (MGR) was also measured in 4-fold and expressed relative to the tester strain.
